# Clinical, electromyographic, and biophysical characterization of the rare Nav1.4 channel mutation *SCN4A* L1436P

**DOI:** 10.3389/fphys.2025.1617672

**Published:** 2025-07-25

**Authors:** François Charles Wang, Olivier Bouquiaux, Isabelle Lievens, Bernard Lakaye, Laura Vandries, Margaux Poleur, Samira Abdou Ide, Nurcan Inci, Vincent Seutin

**Affiliations:** ^1^Clinical Neurophysiology Department, Centre Hospitalier Universitaire de Liège, Liège, Belgium; ^2^Laboratory of Neurophysiology, GIGA Institute, University of Liège, Liège, Belgium; ^3^PRM department, Centre Neurologique et de Réadaptation Fonctionnelle, Neurologic Center, Centre Hospitalier Universitaire de Liège, Fraiture, Belgium; ^4^Neurology Department, Centre Hospitalier Universitaire de Liège, Liège, Belgium; ^5^Molecular Regulation of Neurogenesis, Groupe Interdisciplinaire de Génoprotéomique Appliquée Institute, University of Liège, Liège, Belgium

**Keywords:** case report, myotonia, paramyotonia, SCN4A gene, whole-cell patch-clamp

## Abstract

**Introduction:**

Our aims were to provide an integrated clinical and biophysical characterization of the rare variant NM_000334.4(SCN4A) c.4307T>C (p.Leu1436Pro; L1436P), affecting the skeletal muscle sodium channel Nav1.4, and to compare its functional consequences with both wild-type (WT) channels and the well-established pathogenic variant p.Arg1448His (R1448H).

**Methods:**

We retrospectively analyzed nine unrelated patients carrying the heterozygous L1436P variant. Clinical evaluation included neurological examination and standardized electrodiagnostic protocols. Electromyographic studies were performed at baseline and after muscle cooling. In addition, the biophysical characteristics of the mutant channels were compared to those of WT channels and R1448H, using whole-cell patch clamp recordings of hNav1.4 currents in stably transfected HEK293 cells. Recordings were performed at near physiological temperature (32°C), room temperature (22°C) and cold temperature (15°C).

**Results:**

The clinical phenotypes associated with this SCN4A mutation included sodium channel myotonia (SCM) (case 1), paramyotonia congenita (cases 2, 3, 4, 5, 7 and 8), and cold-aggravated myotonia (case 6) (case 6). Regarding the phenotype of hyperkalemic periodic paralysis, three probands described episodes of muscle weakness (cases 2, 4 and 9). Whole-cell recordings allowed to pinpoint the biophysical defects of L1436P. Thus, this mutation induced a significant slowing down of fast inactivation of the Nav current at several voltages, but this effect was less marked than in R1448H. The L1436P mutation also tended to induce a right shift in the steady-state inactivation curve, but only at cold temperature. On the other hand, a leftward shift in the activation curve was seen at cold and room temperatures with R1448H, but not L1436P. Recovery from fast inactivation was slowed down in both mutants, but only at cold temperature.

**Discussion:**

Our study confirms that the SCN4A-L1436P mutation can give rise to a spectrum of clinical presentations. Epigenetic alterations, modifying genes or environmental factors may influence clinical expression. Our experimental data indicates a relatively milder biophysical phenotype for L1436P as compared to R1448H, which becomes more pronounced at lower temperatures, consistent with the clinical phenotype of a majority of patients.

## Introduction

Sodium channel myotonia (SCM) and *paramyotonia congenita* (PMC) are rare non-dystrophic muscle disorders with muscle hyperexcitability caused by gain-of-function mutations in the voltage-gated skeletal muscle sodium channel gene (*SCN4A*) (Stunnenberg et al., 2020). Clinically, they manifest as myotonia, delayed muscle relaxation after voluntary contraction, resulting in stiffness, pain, fatigue and weakness. SCM is characterized by myotonia at the beginning of muscle activity, which improves with repeated muscle effort (warm-up phenomenon), whereas in PMC, originally described by Eulenburg, myotonia worsens with repeated muscle activity (paradoxical myotonia) and with cold (Eulenburg, 1886). The precise reason why PMC mutations induce symptoms in these specific conditions is so far unclear.

Non-dystrophic myotonias (NDMs) due to *SCN4A* mutations have autosomal dominant inheritance. About a hundred mutations of the gene are described in the literature (Morales and Pusch (2020). These mutations are responsible for the PMC or SCM phenotypes. Among them, the variant c.4307T>C (p.Leu1436Pro or L1436P) has rarely been reported in the literature (Matthews et al., 2008; Bissay et al., 2011). In a recent retrospective epidemiological study on the incidence and prevalence of NDMs in France, the L1436P mutation was found in only eight index cases (out of 1,005 patients) and eleven relatives (out of 1,624 relatives) (data not yet published, but reported as an abstract at the “23^es^ Journées Francophones d’ElectroNeuroMyographie” by Sternberg et al. (2024).

**FIGURE 1:**
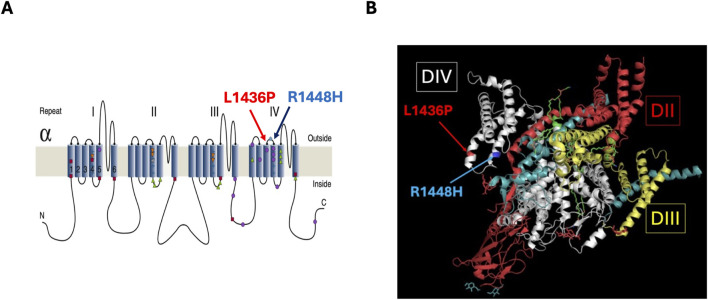
Location of the L1436P and R1448H variants **(A)** 2D representation of α-Subunit of Nav1.4 (Jurkat-Rott et al., 2010). **(B)** 3D representation of human Nav1.4-β1 complex based on cryo-EM structure (Research Collaboratory for Structural Bioinformatics Protein Data Bank).

We retrospectively report the clinical and electrophysiological data of nine apparently unrelated families (nine probands) seen in the Clinical Electrophysiology Department of the University Hospital of Liège (Belgium) between 2005 and 2023, all carrying the c.4307T>C mutation of the *SCN4A* gene. Furthermore, to clarify the functional and cold-related deficit associated with this mutant, we report results from an *in vitro* electrophysiological study (whole-cell patch-clamp) conducted on HEK-293 (Human Embryonic Kidney) cells transfected with the L1436P mutant. The properties of this channel were compared to those of the NM_000334.4(SCN4A):c.4343G>A (p.Arg1448His) mutation (R1448H), which is more commonly found in PMC patients (Jurkat-Rott et al., 2010; Sasaki et al., 2020), as well as those of wild type (WT) channels (Figure 1).

## Methods

### Clinical and electrophysiological data (report of cases)

**FIGURE 2:**
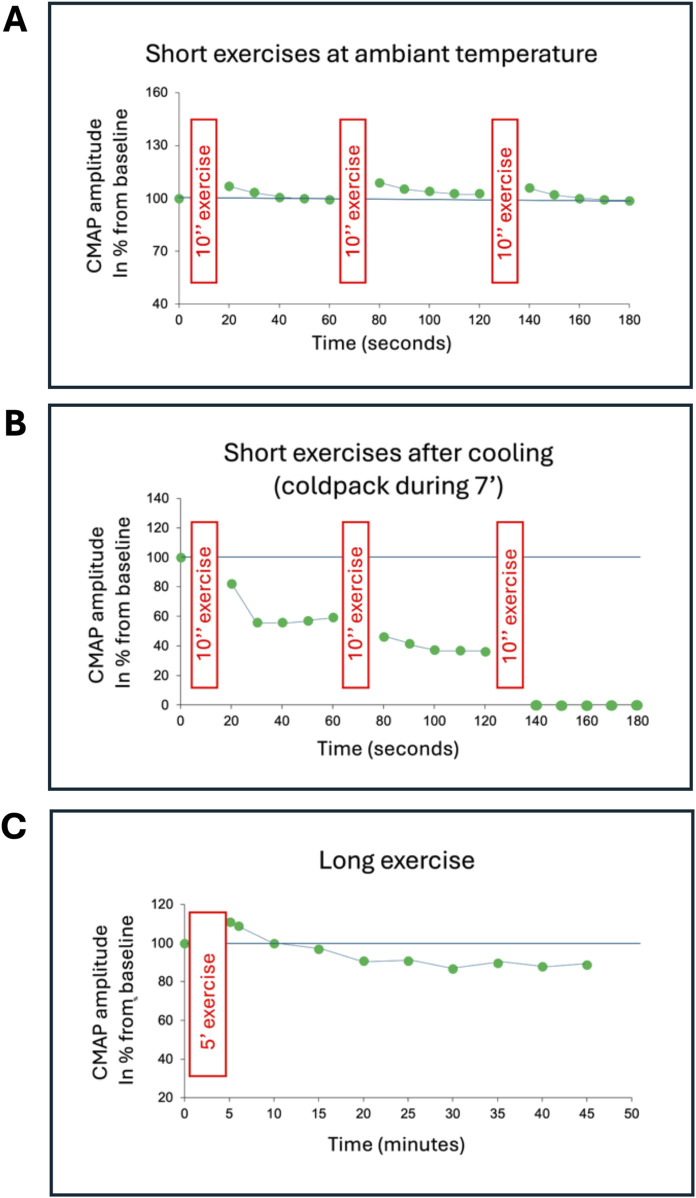
Compound muscle action potential (CMAP) amplitude related to time after short and long exercises (Case 3). The CMAP of the abductor digiti minimi muscle is recorded following stimulation of the ulnar nerve at the wrist. **(A)** The short exercises consist of a maximal voluntary muscle contraction against resistance for 10 s (represented by the red rectangles), repeated three times at 1-min intervals. Prior to this, the supramaximal intensity of nerve stimulation is determined by setting it at 150% of the intensity at which the CMAP amplitude no longer increases. The first 10-s exercise is performed, and the CMAP amplitude is measured immediately after this effort and then four more times every 10 s. The CMAP amplitude is normalized (%) relative to the maximal CMAP amplitude established before the exercises. The same sequence is repeated twice. **(B)** The same test is repeated on the same muscle after 7 min of muscle cooling (cold pack). **(C)** The long exercise is performed on the other abductor digiti minimi muscle to avoid interference from the short exercises and muscle cooling. This exercise consists of a maximal voluntary muscle contraction against resistance for 25 s, followed by 5 s of rest, repeated 10 times (represented by the red rectangle). The CMAP amplitude is measured immediately after this effort, then after 1 min, 4 min later, and then every 5 min for at least 35 min. The CMAP amplitude is normalized (%) relative to the maximal CMAP amplitude established before the long exercise. In this example, during the exercises performed at room temperature **(A)**, the CMAP amplitude slightly increases immediately after each short exercise and then returns to its baseline value, which is most often observed in healthy controls. This slight increase in amplitude reflects post-exercise potentiation due to the excess acetylcholine in the synaptic clefts. After muscle cooling **(B)**, the CMAP amplitude collapses, and this effect becomes more pronounced as the short exercise is repeated, to the point that after the third short exercise, the CMAP amplitude can no longer be reliably measured. During the long exercise **(C)**, the changes in CMAP amplitude are those typically observed in a healthy control, namely, a slight increase in amplitude immediately after the long effort and a moderate and progressive reduction afterward, not exceeding 40% compared to the motor response elicited immediately after the long effort.

The medical history and neurological examination of the nine probands are reported (Cases 1–9). A standard needle-electrode electromyography was performed for each of these patients. The muscles, typically the *anterior tibialis*, quadriceps, common extensor of the fingers, and deltoid, were studied both at rest and during increasing efforts of voluntary contraction. For seven of them (Cases 1–7), these data were completed by an electrophysiological evaluation according to the recommendations of Fournier (Figure 2) (Fournier et al., 2004). The amplitude variations of the CMAP were analyzed both after a long exercise (5 min) and after short exercises (10 s) repeated three times (at 1-min intervals) at room temperature and after 7 min of muscle cooling (Fournier et al., 2006). Furthermore, for cases 1–7, repetitive stimulation at 10 Hz for 10 s of the ulnar nerve at the wrist, with recording from the *abductor digiti minimi* muscle, was performed to document a possible decrement characteristic of certain muscle channelopathies (Michel et al., 2007).

### Genetic analysis data

The nine probands benefited from a polymerase chain reaction (PCR) amplification and sequencing of the 24 exons and parts of the flanking introns of the *SCN4A* gene.

### Whole-cell patch-clamp experiments

Experiments were performed on stable Flp-In™ T-Rex™ 293 cell lines co-expressing SCN4A and SCN1B. Plasmid pcDNA3.1 bearing the human SCN4A cDNA ORF tagged at the c-terminus by a FLAG epitope was obtained from Genscript (clone OHu27269). The whole coding sequence (SCN4A + Flag) was subcloned into pcDNA5/FRT/TO (Invitrogen, Waltham, MA). A DNA fragment comprising the T2A self-cleaving peptide and the human SCN1B ORF (coding for the b1 subunit of the channel and provided by Prof. S.C. Cannon, UCLA, United States) was inserted downstream of the SCN4A-FLAG to allow the simultaneous production of the alpha and beta subunits of the Nav1.4 channel. Mutations were introduced in SCN4A sequence by using the Q5 Site-Directed Mutagenesis kit (New England Biolabs, Massachusetts, United States). Sequencing of all constructs was used to confirm the accuracy of the WT channels and of the mutants that were created. For stable cell line generation, the pOG44 plasmid (Invitrogen) was co-transfected with pcDNA5/FRT/TO co-expressing of Nav1.4 α and β1 subunits and stable clones were selected based on their hygromycin resistance as described by the manufacturer. To induce channel expression, Flp-In™ T-Rex™ 293 cell lines were incubated for 24 h with 1 μg/mL tetracycline before patch-clamp experiments. For the patch clamp experiments, the composition of the extracellular solution, which was superfused at 2 mL/min, was (in mM): 145 NaCl, 4 KCl, 2 CaCl_2_, 1 MgCl_2_, 5 glucose and 10 HEPES, with the pH adjusted to 7.2–7.4 using NaOH. The composition of the internal solution was (in mM): 120 CsF, 10 CsCl, 10 NaCl, 5 EGTA, 5 HEPES, pH adjusted to 7.3 with CsOH. The osmolarity of internal and extracellular solutions was ∼290 and ∼305 mOsm/L, respectively.

Experiments were run at various temperatures using an Accel 500 LC temperature controller from Thermo Fisher Scientific (Waltham, MA, United States) coupled to a home-made heat exchanger. The accuracy of the temperature was checked at the beginning and at the end of each experiment by measuring the temperature in the dish. It was within 0.5°C of the expected value. We used temperatures of 15 (cold), 22 (room temperature, RT) and 32°C (near physiological). Experiments at different temperatures were carried out in different cell samples.

Whole-cell patch-clamp experiments were performed using classical methods. Data was acquired using a Multiclamp 700B amplifier and PClamp 11.1.0.23 software (both from Molecular Devices, Winnersh, England). Low-resistance pipettes (1–3 MΩ) were pulled from filamented borosilicate glass tubing (2.0 mm outer diameter, 0.42 mm wall thickness; Hilgenberg, Malsfeld, Germany) with a P87 puller (Sutter Instruments, Novato, CA, United States). In order to optimize voltage control of fast Na_v_ currents, electrode shanks were coated with wax (H00827, Coltène/Whaledent, Cuyahoga Falls, OH, United States) prior to filling. After break-in, the cell was allowed to stabilize for ≥10 min. Access resistances were below 6 MΩ in most cases and varied by less than 20% during the experiments. Quality control was performed by inspecting the traces during the first activation protocols. We selected recordings where a progressive increase in speed of activation and inactivation occurred between −40 and 0 mV, as expected from the theory. We also excluded cells which had an input resistance below 400 MΩ. Liquid junction potentials and series resistance were not corrected for. First a classical activation protocol was used, starting at −120 mV and depolarizing the membrane during 30 ms in 10 mV increments until +30 mV. For steady-state inactivation, the membrane was maintained at various voltages between −120 and 0 mV during a step of 100 ms, immediately followed by a test pulse to 0 mV. This allowed us to measure the fraction of channels available for activation at different voltages (as compared to −120 mV). We also measured recovery from fast inactivation as follows: after an initial 30 ms pulse from −120 to 0 mV, the membrane was repolarized to −120 mV for various durations, followed by a test pulse to 0 mV. This allowed us to assess the kinetics of recovery from inactivation by dividing the amplitude of the current obtained during each test pulse by the amplitude of the current evoked by the initial pulse.

### Data analysis

Data analysis and curve fitting were performed using Python (version 3.12.7) within a Jupyter Notebook environment and Stimfit 0.15 (Guzman et al., 2014). Inactivation and activation curves were fitted with classical Boltzmann-type equations, allowing to extract k (the slope factor) and V_50_. For activation, current-voltage plots were converted to conductance-voltage plots using the equation G = I/(V-E_Na_), the reversal potential for Na^+^ being + 69 mV at RT in our conditions. In this analysis we considered the values between - 120 and + 30 mV, because values at voltages close to E_Na_ tended to introduce too much variability. Fast inactivation was analyzed by fitting a monoexponential to the data, allowing us to extract the time constant τ. Fits were considered acceptable when R was >0.98.

### Immunofluorescence experiments

To assess the expression of Na_v_1.4 alpha subunits, immunofluorescence assays were carried out. The experiments were performed on four cell cultures: (1) untransfected Flp-In™ T-Rex™ 293 cells, Flp-In™ T-Rex™ 293 cell lines transfected with *SCN4A* and *SCN1B* containing either (2) wild-type, (3) L1436P or (4) R1448H variants of the alpha subunit. For each group, half of the coverslips were exposed for 24 h to 1 μg/mL tetracycline before running the experiment and half were not, giving a total of eight conditions.

Glass coverslips in a 24 well-plate were coated with a Poly-L-ornithine solution (Sigma P4957) for 2 hours at 37 °C and 30,000 cells were seeded in a 70 µL droplet of medium (DMEM high glucose +10% decomplemented fetal bovine serum + penicillin/streptomycin). Once cells were attached, the corresponding medium was supplemented to 1 mL per well and cells were stored in an incubator (37°C, 5% CO2). Twenty-four hours after plating, cells were washed with Dulbecco’s phosphate buffered saline (DPBS), fixed during 10 min with cold methanol and then washed 3 × 5 min with DPBS.

For immunofluorescence, cells were permeabilized and blocked during 30 min at room temperature with phosphate buffered saline (PBS)-triton (0.1%) + 5% bovine serum albumin (BSA) and next incubated 1 hour at room temperature with the primary antibody (Cell Signaling DYKDDDDK Tag (D6W5B) Rabbit mAb #14793) diluted 1:100 in PBS-triton (0.1%) + 5% BSA. Next, they were washed 3 × 5 min with PBS 0.1M and incubated 1 h at room temperature in a solution containing the secondary antibody (Goat anti-Rabbit IgG, H + L, Cross-Adsorbed Secondary Antibody, coupled to Alexa Fluor™ 568) (Invitrogen A-11011) diluted 1:500 in PBS-triton (0.1%) and supplemented with DAPI (Sigma D9542) at 1 μg/mL. Finally, coverslips were washed 3 × 5 min with PBS 0.1M and mounted on slide with an Aqua-poly mount mounting medim (Polysciences 18606).

Image analysis was performed as follows. Images were acquired using a Leica SP5 inverted confocal microscope. Acquisitions were performed with a Plan-Apochromat 40×/1.3 oil immersion objective, with an image resolution of 512 × 512 pixels, a pixel size of 0.37 µm in X and Y, a Z-step size of 1.98 µm, and scan speed of 400 Hz. The flag-tagged channel was visualized using a 561 nm laser set at 44%, with an AOBS detection window between 563 and 695 nm, and a gain of 394 V. DAPI staining was acquired using a 405 nm laser set at 21%, with an AOBS window between 410 and 450 nm, and a gain of 632 V. Z-stacks were projected using sum intensity projections to generate quantifiable 2D images, which were processed with a custom-made script in FIJI (v1.54). Cell detection was subsequently performed using the “Cell Detection” function in QuPath (v0.5.1) with the following parameters: background subtraction radius of 15 μm, cell expansion of 2.5 µm around nuclei, Gaussian blur of 2.5, intensity threshold set to 10, and segmentation using a watershed algorithm. Fluorescence intensity of the flag-tagged channel was then measured in each cell, and data were exported for further analysis. Depending on the availability of the material, 2 to 5 pictures were taken on two replicate coverslips for each condition (see above the eight conditions).

The threshold applied was the maximal mean fluorescence intensity (MFI) (background) detected in empty Flp-In™ T-REx™ (35.56 for Flp-In™ T-REx™ + tetracycline and 15.06 for Flp-In™ T-REx™ without tetracycline), meaning that the MFI of all cells ≤ these values was considered as 0.

### Statistical Analysis

Statistical analysis was performed using SAS software (SAS University Edition, Cary, NC). Descriptive statistics are reported as medians and interquartile ranges (IQR). Given the small and variable sample sizes, only non-parametric tests were used: the Kruskal-Wallis (KW) test for comparisons of more than two groups; if the KW showed a significant difference between the various groups, *post hoc* pairwise comparisons were performed using the Dwass, Steel, Critchlow-Fligner (DSCF) method; the effect size for the KW test was measured using *eta* ^2^ = (H−k+1)/(n−k), where H = KW test statistic, k = number of groups, and n = total number of observations (≥0.14: large effect); and the effect size r for DSCF was calculated by dividing the Z statistic (obtained from the Wilcoxon test) by the square root of the total number of observations (≥0.50: large effect). For immunofluorescence experiments, a Dunn’s *post hoc* test was applied. A p-value <0.05 was considered statistically significant.

## Results

### Report of cases

Genetically, the nine cases exhibited, in the heterozygous state, the missense mutation L1436P in exon 24 of the *SCN4A* gene, changing a highly conserved leucine to a proline. Case 9 was unique in presenting, in addition to the L1436P mutation, a c.5211dup (p.Tyr1738LeufsTer27) duplication of the *SCN4A* gene of indeterminate significance. The L1436P mutation was also found in six relatives of four probands (a niece and a daughter of case 6, the mother of cases 3 and 5, and 2 daughters of case 1).

Table 1 presents the clinical and electrophysiological data regarding the nine probands with the L1436P mutation.

**TABLE 1:** clinical and electrophysiological findings of the 9 probants with L1436P variant.

	Case 1	Case 2	Case 3	Case 4	Case 5	Case 6	Case 7	Case 8	Case 9
Gender	female	female	female	male	female	female	female	female	female
Age at onset (year)	36	34	36	20	46	60	37	66	50
Clinical myotonia	Yes	Yes	Yes	Yes	Yes	Yes	Yes	Yes	Yes
- History suggestive	Yes	Yes	Yes	Yes	Yes	Yes	Yes	Yes	No
- Mechanical myotonia	Yes	Yes	Yes	Yes	Yes	Yes	Yes	Yes	Yes
Episodic weakness	No	Yes	No	Yes	No	No	No	No	Yes
Warm-up phenomenon	Yes	No	No	No	No	No	No	No	No
Exacerbation during exercise	No	NA	Yes	Yes	No	NA	Yes	Yes	Yes
Exacerbation after exercise	NA	Yes	Yes	No	NA	NA	No	No	No
Cold exacerbation	No	Yes	Yes	Yes	Yes	Yes	Yes	Yes	No
Stress exacerbation	NA	NA	NA	Yes	NA	NA	NA	NA	NA
Pain	Yes	Yes	Yes	Yes	No	Yes	No	NA	Yes
- Severity	- moderate	- moderate	- mild	- mild		- mild			- moderate
- Distribution	- arms, hands, feet, thighs	- hands, thighs, calves	- diffuse	- face, hands		- hands			- lower > upper limbs
Muscle bulk	Hypertrophy^a^	Normal	Normal	Hypertrophy^b^	Normal	Normal	Normal	Normal	Normal
Myotonic discharges on needle EMG	Yes	Yes	Yes	Yes	Yes	Yes	Yes	Yes	Yes
Myogenic traces	No	No	No	No	No	No	No	No	Yes
CMAP amplitude									
- After short exercise	- No decrease	- No decrease	- No decrease	- No decrease	- No decrease	- No decrease	- No decrease	ND	ND
- Idem after cooling	- No decrease	- Decrease	- Decrease	- Decrease	- Decrease	- No decrease	- Decrease		
- After long exercise	- No change	- No change	- No change	- No change	- No change	- No change	- No change		
- Postexercise myotonic potential	- No	- No	- No	- No	- No	- No	- No		
10 Hz decrement	No	No	No	No	No	No	No	ND	ND

EMG, electromyography; CMAP, compound muscle action potential; NA, not applicable; ND, not done.

^a^ Pseudo-athletic aspect.

^b^ Calf hypertrophy.

The sex was predominantly female (8 out of 9 cases). All six related cases were also female. The age of onset of symptoms ranged from 20 to 66 years (average 42.8; standard deviation: 14.2).

The nine cases exhibited clinical myotonias: either patients reported episodes of muscle stiffness and rigidity (8 out of 9 cases) during their medical history, or mechanical myotonias were identified upon *extensor digitorum* muscle percussion during the clinical examination (9 out of 9 cases), see the Supplementary Video S1. Only cases 2, 4 and 9 reported episodes of muscle weakness. Muscle pain was frequently reported (6 out of 9 cases). The intensity of this pain was either mild (cases 3, 4, 6) or moderate (cases 1, 2, 9), but never severe. The distribution of this pain was either diffuse (cases 3, 9) or more localized, with pain reported in the hands in four cases (1, 2, 4, 6), thighs in two cases (1, 2), arms and feet in case 1, and calves in case 2. Except for cases 1 and 9, symptoms were exacerbated by exposure to cold. Only case 1 reported a warm-up phenomenon, a feature of SCM, not PMC. The exacerbation of symptoms was often related to exercise (6 out of 8 cases), either during the repetition of the exercise (cases 3, 4, 7, 8, 9), or at rest after exercise (cases 2, 3). Only case 4 spontaneously reported an exacerbation of symptoms related to stress. Muscle trophicity was mostly normal (7 out of 9 cases). Case 1 had a pseudo-athletic appearance, while case 4 presented with calf hypertrophy.

Electrophysiologically, the nine cases had very abundant myotonic discharges, characteristic “dive bomber” spontaneous discharges, during the needle-electrode examination of the skeletal musculature. The electromyographic (EMG) recordings did not show any specific abnormalities, except for case 9 where myopathic-like traces were recorded, with EMG traces reduced in amplitude and too early interference pattern during right quadriceps muscle contraction of increasing intensity. The study of the amplitude variation of the compound muscle action potential (CMAP) of the *abductor digiti minimi* muscle following repetitive nerve stimulation of the ulnar nerve at 10 Hz never revealed a significant decrement at room temperature. For cases 1–7, the CMAP amplitude was never significantly reduced after short exercises performed at room temperature. No significant change in CMAP amplitude was observed after a long exercise. No post-exercise myotonic potentials were recorded. However, after muscle cooling, the CMAP amplitude was significantly reduced after short exercises in cases 2, 3, 4, 5, and 7, with the amplitude reduction becoming more pronounced with each repetition of the short exercise (Figure 2).

### Biophysical data

To evaluate the degree to which the Nav channel function was altered in our patients, we carried out experiments in transfected HEK293 cells (see methods). To have relevant comparison points, we studied WT, L1436P and R1448H channels. The reason for choosing the latter is that it is one of the most frequent variants (Sasaki et al., 2020). As a first step, we evaluated the expression of the different channel variants in stable cell lines using immunofluorescence (see Methods). The results revealed the expression of the Na_v_ channels in all three variants with tetracycline induction. As expected, untransfected cells yielded no signal. This is represented in Supplementary Figures S1 and S2 and in Supplementary Table S1.

**FIGURE 3:**
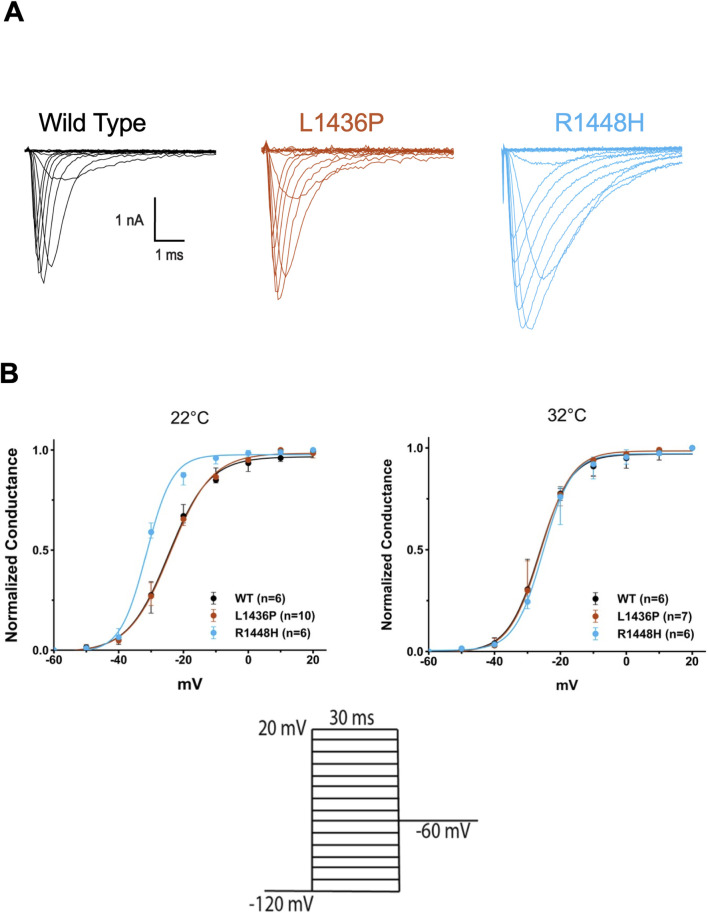
Activation and fast inactivation of L1436P, R1448H and WT channels **(A)** Raw traces of Nav currents that were recorded at 22°C in HEK293T cells expressing WT or mutant hNav1.4 channels. One representative cell is shown for each subtype. Ten mV steps from −120 to 30 mV evoked a rapidly inactivating current with slower inactivation kinetics in the mutants (especially R1448H). Traces have been leak-subtracted (see Methods) **(B)** Normalized conductance (G/Gmax) versus voltage curves obtained for L1436P (n = 10); R1448H (n = 6), and WT channels (n = 6) using the equation G = I/(V-Vrev), with Vrev being +69 mV under our conditions). R1448H channels activated at more negative potentials than the other species at 22°C, but not at 32 °C. Bars represent interquartile range. The voltage commands are shown below.

Due to the cold sensitivity of many of the patients’ symptoms, we performed patch clamp experiments at three temperatures: cold (15°C), room temperature (RT) (22°C) and near physiological (32°C). We first studied the activation of the channels with a classical protocol starting from - 120 mV. Examples of raw traces for WT and each mutation are shown in Figure 3A. The data was converted to conductance-voltage curves (see Methods) and the voltages of half maximal activation (V_50_) were extracted. Two examples of such curves are displayed in Figures 3B,C and the numerical data is provided in Table 2. The L1436P mutant activated at the same voltages as the WT channels, whereas the R1448H mutant activated at significantly more negative voltages in cold and room temperature, but not at 32°C (see Tables 2, 3 for the statistics).

**TABLE 2:** Biophysical parameters of the three types of Nav1.4 channels that were studied.

		15°C			22°C			32°C		
		WT	LP	RH	WT	LP	RH	WT	LP	RH
V_50_ of fast activation (mV)	Median	−32.13 (8)	−30.55 (6)	−38.59 (11)	−24.35 (6)	−24.41 (10)	−31.49 (6)	−25.36 (6)	−25.71 (7)	−25.41 (6)
	IQR	6.15	1.33	6.85	2.88	2.47	1.23	4.12	3.70	2.81
	KW (η^2^)	p = 0.0017 (0.49)			p = 0.0052 (0.45)			p = 0.5714		
Time constant of fast inactivation at -10 mV (ms)	Median	0.93 (8)	1.76 (5)	4.42 (11)	0.41 (6)	0.69 (9)	1.76 (6)	0.18 (6)	0.33 (7)	0.95 (6)
	IQR	0.11	0.37	0.46	0.03	0.16	0.46	0.03	0.03	0.11
	KW (η^2^)	p < 0.0001 (0.85)			p = 0.0002 (0.86)			p = 0.0005 (0.83)		
V_50_ of steady-state inactivation (mV)	Median	−70.17 (7)	−59.96 (6)	−67.37 (11)	−59.82 (5)	−54.10 (10)	−58.95 (6)	−52.11 (6)	−51.29 (7)	−54.55 (6)
	IQR	7.36	2.59	3.99	2.96	3.02	3.16	7.93	2.88	7.45
	KW (η^2^)	p = 0.0152 (0.30)			p = 0.0057 (0.46)			p = 0.9806		
Time constant of recovery from fast inactivation at -120 mV (ms)	Median	3.07 (8)	1.58 (5)	2.19 (10)	0.64 (6)	0.47 (9)	0.66 (6)	0.36 (6)	0.39 (7)	0.33 (6)
	IQR	0.56	0.44	0.66	0.28	0.24	0.13	0.09	0.23	0.15
	KW (η^2^)	p = 0.0005 (0.67)			p = 0.1693			p = 0.5461		

WT, wild type; LP = L1436P mutant; RH = R1448H mutant. Values are represented as median and interquartile range (IQR). The number of experiments is indicated in brackets. Statistically significant differences between WT and mutant channels (L1436P and R1448H) were determined using a Kruskal-Wallis (KW) analysis. Effect size for the KW test is measured by eta2 (η2) = (H−k+1)/(n−k) with H = KW test statistic, k = number of groups, n = total number of observations (≥0.14: Large effect).

**TABLE 3:** Post-hoc pairwise comparisons by the Dwass, Steel, Critchlow-Fligner method.

		15°C	22°C	32°C
V_50_ of fast activation (mV)	WT versus LP	p = 0.9207	p = 0.9935	p = 0.9972
	WT versus RH	p = 0.0083 (0.67)	p = 0.0281 (0.72)	p = 0.7026
	LP versus RH	p = 0.0100 (0.69)	p = 0.0068 (0.75)	p = 0.5766
Time constant of fast inactivation at -10 mV (ms)	WT versus LP	p = 0.0096 (0.79)	p = 0.0042 (0.81)	p = 0.0182 (0.73)
	WT versus RH	p = 0.0008 (0.82)	p = 0.0110 (0.81)	p = 0.0110 (0.81)
	LP versus RH	p = 0.0052 (0.76)	p = 0.0042 (0.76)	p = 0.0076 (0.81)
V_50_ of steady-state inactivation (mV)	WT versus LP	p = 0.1122	p = 0.0604	p = 0.9888
	WT versus RH	p = 0.7216	p = 1.000	p = 0.8806
	LP versus RH	p = 0.0100 (0.69)	p = 0.0095 (0.72)	p = 0.9037
Time constant of recovery from fast inactivation at -120 mV (ms)	WT versus LP	p = 0.0096 (0.79)	p = 0.4660	p = 0.7550
	WT versus RH	p = 0.0053 (0.72)	p = 0.9860	p = 0.9860
	LP versus RH	p = 0.0521	p = 0.1428	p = 0.4877

WT, wild type; LP = L1436P mutant; RH = R1448H mutant. Effect size r (in brackets) is calculated by dividing the Z statistic (obtained from the Wilcoxon test) by the square root of the total number of observations (≥0.50: Large effect).

**FIGURE 4:**
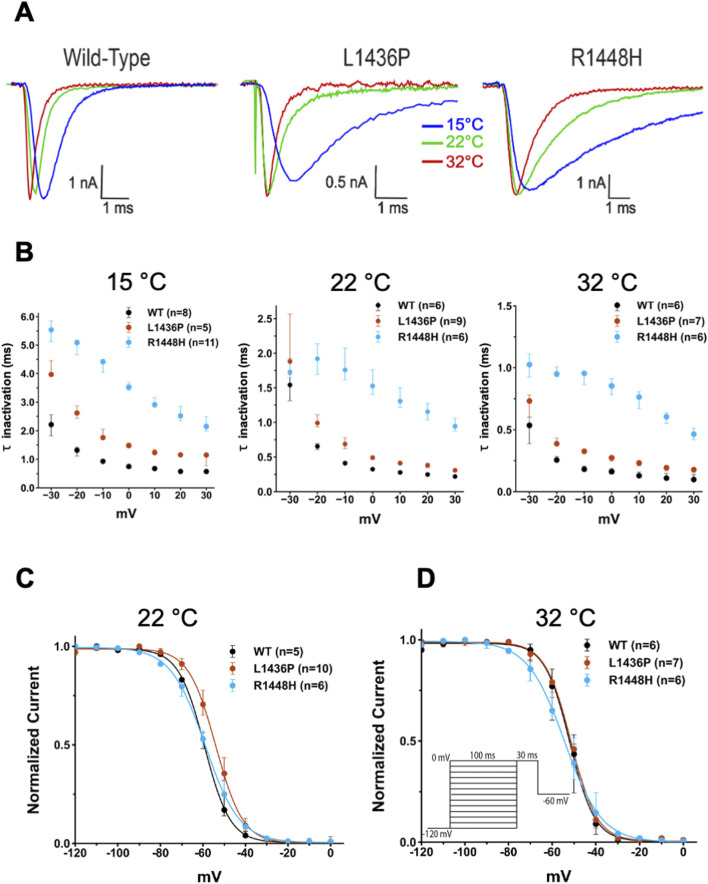
Effect of temperature on fast inactivation of the various channels and steady-state inactivation **(A)** Representative raw traces recorded at −10 mV of WT, L1436P, and R1448H currents at 15°C, 22°C and 32°C. The amplitudes of the currents have been scaled to the same maximal amplitude to better compare the channel kinetics. Note the marked slowing down of fast inactivation (and of activation) in the three channel species when temperature was lower. **(B)** Summary of voltage- and temperature-dependence of inactivation time constants. The decay of the current at depolarized voltages was best described by a single exponential fit, with the resulting time constants (τ) plotted as a function of voltage. Note the differences in the vertical scales at the three temperatures. The voltage command is identical to that in Figure 3. **(C)** and **(D)** The steady-state inactivation curve of the L1436P mutant was slightly more depolarized than the one of the other species at 22°C, but not at 32°C. The voltage command is inserted in panel **(D)**. In **(B)**, **(C)** and **(D)**, bars represent interquartile range.

We next turned our attention to the fast inactivation of the channels following their activation. The R1448H mutant is known to have a much slower inactivation than WT channels and this feature is considered as the main contributing factor to the induction of myotonic runs (Chahine et al., 1994; Richmond et al., 1997). We confirmed this observation, as shown in Figures 3A, 4A,B. Indeed, values of the time constant of inactivation were much larger in R1448H than in WT (Table 3; Figures 4A,B). For example, at −10 mV, the median value was 0.95 ms in R1448H and 0.18 ms in WT at 32°C and remained much larger in R1448H throughout temperatures. The L1436P mutant’s behavior was intermediate in this respect, with a value of 0.33 ms at the same voltage at 32°C. The time constant became significantly higher at lower temperatures in all genotypes (Figures 4A,B; Table 2). At 22°C, which corresponds to moderate cold, values were 0.41, 0.69 and 1.76 ms for WT, L1436P and R1448H, respectively. Overall, the L1436P mutant inactivated significantly slowlier than the WT. However, its effect was also significantly smaller than the one of R1448H across all voltages and temperatures (see Figure 4B; Table 3 for pairwise comparisons).

One other important aspect of Na_v_ function is the recovery from inactivation upon repolarization of the membrane. This parameter was also evaluated and showed a faster recovery in the L1436P and R1448H mutants as compared to the WT channels, but only at cold temperature (see Tables 2, 3).

Steady-state inactivation, which reflects the availability of the channels at various voltages, was studied next. For this purpose, the membrane was clamped for 100 ms at various voltages before giving a pulse to 0 mV. The amount of current measured at this voltage reflects the percentage of channels that are not inactivated. The fitting of the curves allowed us to extract the V_50_ of steady-state inactivation (see Methods). The L1436P mutant significantly differed from the R1448H mutant in terms of steady-state inactivation (Figure 4C; Table 3), but not from the WT channels, although a trend was noted (p = 0.06 at 22°C). Thus, the V_50_ was more depolarized in the L1436P mutant than in the R1448H mutant, both at RT and cold temperatures (by 4–7 mV), but not at near physiological temperature (Figure 4D). The consequence of this is that a higher percentage of L1436P channels are available in the region of the resting membrane potential of myocytes (∼- 85 mV) than is the case for the R1448H mutant.

## Discussion

The L1436P mutation was first mentioned in 2008 by a London team (Matthews et al., 2008). The case reported by these authors presented a typical clinical phenotype of *paramyotonia* with myotonias exacerbated by exercise and exposure to cold, as well as muscle pain.

The second time the L1436P mutation was mentioned in the literature was by Belgian neurologists (Bissay et al., 2011), who described this particular variant in 3 unrelated families (3 probands and 8 relatives) from the Brussels region. The clinical phenotype of these patients supported a diagnosis of SCM. Indeed, they met the main criteria for SCM, specifically exercise-induced delayed-onset myotonia (8 out of 11 cases) and a warm-up phenomenon (symptoms relieved by repetitive muscle contraction) without weakness (10 out of 11 cases). Additionally, clinical electrophysiological data were more indicative of SCM than of PMC, as short exercises performed at room temperature and after muscle cooling did not significantly reduce the CMAP amplitude (7 out of 7 cases). Indeed, their electrophysiological data corresponded with Pattern III of the Fournier classification, both at room temperature and after muscle cooling (Fournier et al., 2004; Fournier et al., 2006). However, surprisingly, the authors noted that the phenotype of their families differed from classic SCM in that the myotonia did worsen with cold exposure and the frequency and severity of muscle pain. For these reasons, the authors proposed adding a fourth category to the *fluctuans*, *permanent* and acetazolamide-responsive myotonias, which they named cold-aggravated myotonias.

Therefore, in the original article concerning the L1436P mutant, the described phenotype was PMC (Matthews et al., 2008), and in the second article (Bissay et al., 2011), the reported phenotype in the three families was SCM that worsened with cold. Additionally, the National Center for Biotechnology Information (NCBI) classifies the L1436P variant as hyperkalemic periodic paralysis. The cases described in our work confirm the heterogeneity of the phenotype linked to the L1436P mutation. Indeed, among the seven probands for whom clinical and electrophysiological data were available, the most frequent phenotype was that of PMC (cases 2, 3, 4, 5, and 7), characterized by worsening of symptoms during and/or after exercise—thus without a warm-up phenomenon—worsening of symptoms when exposed to cold, and a Pattern I in electrophysiological evaluation according to Fournier’s protocol (a decrease in the CMAP amplitude that intensifies with the repetition of short exercises, particularly after muscle cooling) (Figure 2). The SCM phenotype was observed only once (case 1), with symptoms appearing at the initiation of movement and improvement of symptoms through repeated exercise (warm-up phenomenon), associated with a Pattern III in the electrophysiological evaluation according to Fournier’s protocol (no significant change in CMAP amplitude during short exercises, including after muscle cooling) (Fournier et al., 2004; Fournier et al., 2006). Case 8 presented clinically as a PMC, but electrophysiological evaluation using Fournier’s protocol was not performed. The phenotype of case 6 was more difficult to identify, as it was only characterized by worsening of symptoms upon cold exposure (symptoms were neither worsened nor improved by exercise, and there was no change in CMAP amplitude during short exercises). This phenotype resembles what Bissay et al. (2011) identified as cold-aggravated myotonia. Regarding the phenotype of hyperkalemic periodic paralysis, three probands described episodes of muscle weakness (cases 2, 4, 9), while the long exercise test, performed for cases 1–7, was negative due to the absence of significant and characteristic changes in CMAP amplitude, either the immediate increase after exercise or the late reduction after exercise (Figure 2) (Fournier et al., 2004). The clinical data confirm that the L1436P mutation is frequently associated with muscle pain, which often constituted the reason for consultation (cases 1, 2, 3, 4, 6, 9) and sometimes led to an initial misdiagnosis of fibromyalgia. Muscle hypertrophy was mostly absent. Nevertheless, case 1 with an SCM phenotype presented with a pseudo-athletic appearance, while case 4 (*paramyotonia* phenotype and the only male in our series) had calf hypertrophy. Thus, we confirm that the L1436P mutation of the *SCN4A* gene can cause different phenotypes. So far unknown epigenetic alterations, modifying genes and/or environmental factors may influence the clinical expression within and between families with the same *SCN4A* mutation. The male-to-female ratio found in this work (only one male among 9 probands and 6 relatives) suggests that hormonal climate could play a role in the phenotypic expression of this genetic disease. This gender-related peculiarity was not reported by Bissay whose cases included 4 women and 7 men (Bissay et al., 2011).

Case 9 was somewhat unusual in that the medical history was not suggestive of myotonia. It was the clinical examination and EMG that indicated a myotonic syndrome by demonstrating clinical myotonia and typical myotonic bursts. The patient complained of muscle pain and weakness unrelated to cold exposure. The EMG recorded myogenic-like features in the right quadriceps muscle. This somewhat unusual presentation may be related to the presence of a second variant of uncertain significance, a c.5211dup (p.Tyr1738LeufsTer27) duplication of the *SCN4A* gene. This duplication causes a frameshift and the creation of a premature stop codon. This truncating mutation would therefore be associated with a loss of function of one of the two alleles. However, this variant is absent from population databases (gnomAD) and clinical databases (ClinVar). Furthermore, we do not know whether the two variants are in trans or in cis. If p.Tyr1738LeufsTer27 and L1436P are in trans, the patient could be a compound heterozygote for SCN4A, with an increased risk of a severe or atypical form of muscle channelopathy.

The 26 reported cases of the L1436P mutation of the *SCN4A* gene in Belgium—specifically, three probands and eight relatives in the Brussels region (Bissay et al., 2011), and nine probands and six relatives in the Liège region—contrast with the limited data available in the literature and with the 17 cases (8 probands and 11 relatives) found in a large epidemiological study by Sternberg (not yet published) on the incidence and prevalence of NDMs in France (a country six times more populous than Belgium). It is therefore possible that there is a founder effect in Belgium related to this mutant.

Our patch clamp results can be summarized as follows: we show that the L1436P mutant induces several abnormalities, including a significant, yet modest slowing down of fast inactivation across several voltages and a mildly depolarized steady-state inactivation curve, the latter meaning a slightly higher availability of channels. On the other hand, voltage-dependence of activation of the channels remains similar to the WT. Overall, its biophysical phenotype seems less severe than the one of the R1448H mutant. In addition, a striking feature of both mutants is that most abnormalities were observed at relatively cold or very cold temperatures (except for the slowing of the fast inactivation). In this regard, our results emphasize the usefulness of recording currents at various temperatures.

Do these biophysical abnormalities explain the myotonic phenotype of the L1436P patients? This is hard to say. First, we have not yet tested all biophysical parameters. For example, some Na_v_ mutants exhibit defects in slow inactivation (Webb and Cannon, 2008; Carle et al., 2009), i.e., an inactivation process that is different from the one that occurs immediately after activation and proceeds over a time scale of seconds or minutes (Hayward et al., 1997). It may be that L1436P differs from WT channels in this respect. In addition, there is a clear need to incorporate these different abnormalities in a realistic myocyte model to be able to make predictions. This will allow us to check whether the modest increases in fast inactivation time constants are sufficient to explain the electromyographic phenotype. We are currently developing a new conductance-based computer model of a myocyte based on the one by Cannon (Cannon et al., 1993) with improved features such as incorporation of recent data on ClC-1 and K_ir_2.1 biophysics, as well as the possibility to include two types of Na_v_ currents, a normal one and a pathological one (thereby mimicking the heterozygosity of the patients). This will hopefully help us to answer this question.

One interesting point is that both clinical and biophysical evaluation of the L1436P mutant shows that it manifests itself predominantly at cold temperatures with overall less alterations at physiological temperature.

## Conclusion

In conclusion, we have described the clinical and biophysical characteristics of a rare Na_v_1.4 mutant, L1436P. The patients bearing this mutation show a rather variable phenotype. Our patch clamp analysis shows biophysical alterations that worsen at infraphysiological temperatures. This is consistent with what was observed clinically in a majority of, but not all our patients.

## Data Availability

The original contributions presented in the study are included in the article/Supplementary Material, further inquiries can be directed to the corresponding author.

## Supplementary material

SUPPLEMENTARY VIDEO mechanical myotonias identified upon extensor digitorum muscle.

SUPPLEMENTARY FIGURE 1 Representative photomicrographs depicting the fluorescence intensity in the wild type (WT), R1448H (RH), L1436P (LP) (induced or not with tetracycline) and the empty Flp-In™ T-REx™cells (with or without tetracycline) as negative control. Note that the color code at the top right of each figure is only relevant for the flag-tagged channel and not for the DAPI staining which is represented in grey without any intensity indication. scale: 50 μm

SUPPLEMENTARY FIGURE 2 Mean fluorescence intensity (MFI) in cells transfected with wild-type (WT), L1436P (LP), and R1448H (RH) channels, as well as in untransfected control cells (FLP). Each condition was analyzed with and without tetracycline (tcy) induction. A highly significant increase in MFI was observed in all three transfected groups following tetracycline treatment (Kruskal–Wallis test followed by Dunn’s post-hoc test; p < 0.0001).

## Glossary

ANOVA: Analysis of Variance
CMAP: Compound Muscle Action Potential
DSCF: Dwass, Steel, Critchlow-Finger method
HEK: Human Embryonic Kidney
KW: Kruskal-Wallis
L1436P: NM_000334.4(SCN4A):c.4307T>C (p.Leu1436Pro)
NDM: Non-Dystrophic Myotonia
PMC: *Paramyotonia Congenita*
R1448H: NM_000334.4(SCN4A):c.4343G>A (p.Arg1448His)
SCM: Sodium Channel Myotonia
*SCN4A*: Sodium Voltage-Gated Channel Alpha Subunit 4
WT: Wild Type
PCR: polymerase chain reaction
IQR: interquartile range
