# Supplementary figures and images for: Clinical, electromyographic, and biophysical characterization of the rare Nav1.4 channel mutation SCN4A L1436P
Source: Front Physiol. 2025 Jul 25;16:1617672. doi: 10.3389/fphys.2025.1617672 (PMC12366458; doi:10.3389/fphys.2025.1617672)

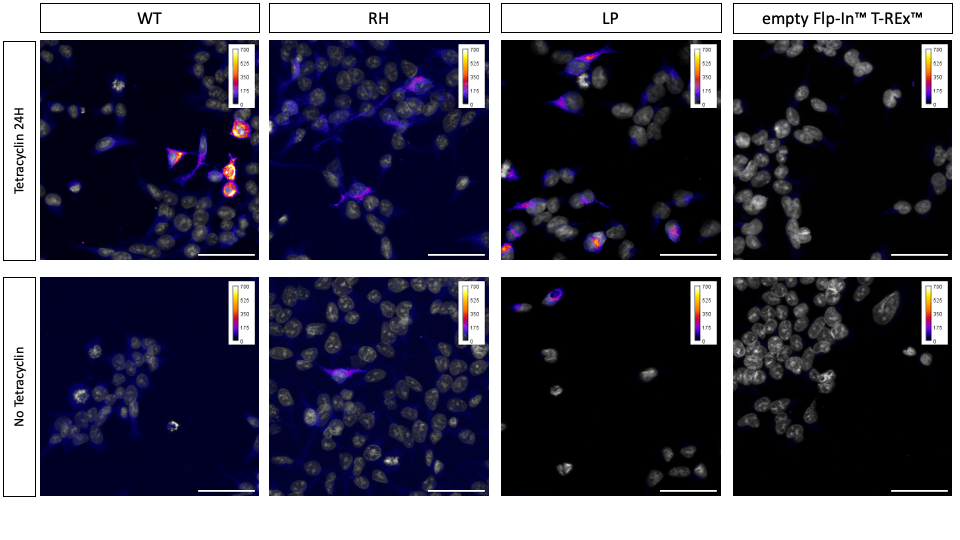

Supplement: Supplementary file 1 [file Image1.tiff]

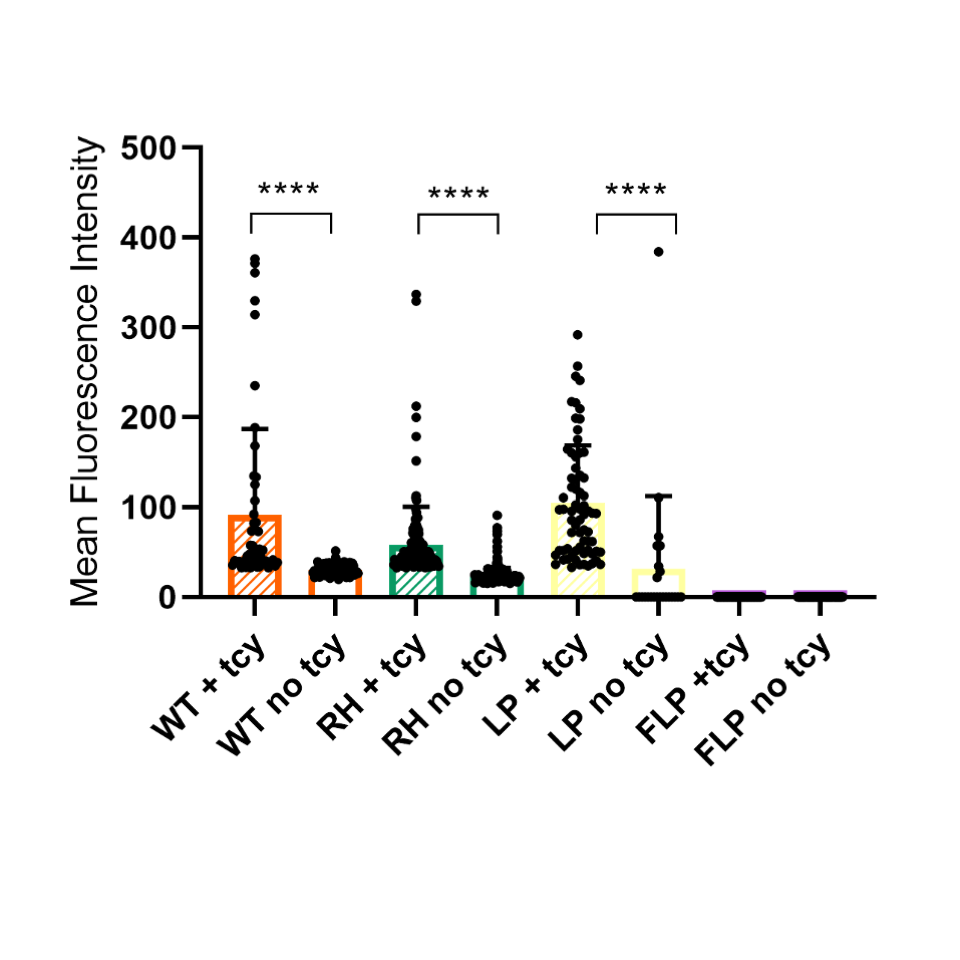

Supplement: Supplementary file 2 [file Image2.tiff]
